# Genome-Wide Association Studies Identifying Multiple Loci Associated With Alfalfa Forage Quality

**DOI:** 10.3389/fpls.2021.648192

**Published:** 2021-06-18

**Authors:** Sen Lin, Cesar Augusto Medina, O. Steven Norberg, David Combs, Guojie Wang, Glenn Shewmaker, Steve Fransen, Don Llewellyn, Long-Xi Yu

**Affiliations:** ^1^Plant Germplasm Introduction Testing and Research, Agricultural Research Service, United States Department of Agriculture, Prosser, WA, United States; ^2^Franklin County Extension Office, Washington State University, Pasco, WA, United States; ^3^Department of Dairy Science, University of Wisconsin, Madison, WI, United States; ^4^Eastern Oregon Agricultural and Natural Resource Program, Oregon State University, La Grande, OR, United States; ^5^Kimberly R&E Center, University of Idaho, Kimberly, ID, United States; ^6^Irrigated Agriculture Research and Extension Center, Washington State University, Prosser, WA, United States; ^7^Department of Animal Sciences, Washington State University, Pullman, WA, United States

**Keywords:** alfalfa, GWAS, markers, forage quality, GBS

## Abstract

Autotetraploid alfalfa is a major hay crop planted all over the world due to its adaptation in different environments and high quality for animal feed. However, the genetic basis of alfalfa quality is not fully understood. In this study, a diverse panel of 200 alfalfa accessions were planted in field trials using augmented experimental design at three locations in 2018 and 2019. Thirty-four quality traits were evaluated by Near Infrared Reflectance Spectroscopy (NIRS). The plants were genotyped using a genotyping by sequencing (GBS) approach and over 46,000 single nucleotide polymorphisms (SNPs) were obtained after variant calling and filtering. Genome-wide association studies (GWAS) identified 28 SNP markers associated with 16 quality traits. Among them, most of the markers were associated with fiber digestibility and protein content. Phenotypic variations were analyzed from three locations and different sets of markers were identified by GWAS when using phenotypic data from different locations, indicating that alfalfa quality traits were also affected by environmental factors. Among different sets of markers identified by location, two markers were associated with nine traits of fiber digestibility. One marker associated with lignin content was identified consistently in multiple environments. Putative candidate genes underlying fiber-related loci were identified and they are involved in the lignin and cell wall biosynthesis. The DNA markers and associated genes identified in this study will be useful for the genetic improvement of forage quality in alfalfa after the validation of the markers.

## Introduction

Alfalfa is so called “The Queen of Forages” due to its high value of nutrients beneficial to livestock performance (Hrbáčková et al., 2020). The quality of alfalfa can directly affect daily animal response and gains (Popp et al., 2000). However, alfalfa quality varied remarkably by genotype, and the genetic basis that influences the quality traits is still poorly understood. The autotetraploid alfalfa and its outcrossing characteristics make it more challenging for genetic gain in the economical traits. Fiber content and digestibility are both major factors that affect alfalfa quality. Acid detergent fiber (ADF) and neutral detergent fiber (NDF) are two important parameters on the estimation of fiber quality in forage. The difference between ADF and NDF is that ADF is part of NDF without the hemicellulose, which can be removed by boiling the hay in a detergent solution in acid conditions (Bonsi et al., 1995). Thus, ADF only contains cellulose, lignin and cutin. Lower ADF value is associated with higher quality of alfalfa (Riday et al., 2002). Since NDF takes hemicellulose into account, which is partially digestible by livestock, it presents the forage amount that can be utilized by livestock, and predicts the energy value of alfalfa. The amount of NDF digested before passing from rumen can be estimated by NDF digestibility (NDFD) in 24–48 h. NDFD in longer times (120–240 h) is used to measure the slow and indigestible components. Recently, an *in vitro* assay was developed for the prediction of total tract NDF digestion (TTNDFD), which is a direct predictor of the performances of cattle when fed with forages that differ in fiber digestibility (Lopes et al., 2015). Other broadly used indexes, such as relative feed value (RFV) and relative forage quality (RFQ) are also related to the fiber quantity and digestibility in alfalfa (Undersander et al., 2002). Both RFV and RFQ have been used for ranking forage quality based on the intake potential and digestible matter.

Lignin is an anti-quality component and its content directly affects forage quality. Genetic improvement has been made in alfalfa to reduce the lignin content. Manipulation of the genes involved in lignin biosynthesis reduced lignin content and improved the digestibility of alfalfa for ruminant animals (Jung et al., 2012). Syringyl (S)-lignin, p-hydroxyphenyl (H)-lignin and guaiacyl (G)-lignin are three basic units of lignin in plants. Of those, G-lignin is the primary component in alfalfa. It was reported that knockdown of caffeic acid 3-O-methytransferse (COMT) reduced G-lignin and total lignin contents (Guo et al., 2001). Down-regulation of P450 enzymes cinnamate 4-hydroxylase (C4H), coumarate 3-hydroxylase (C3H) or ferulate 5-hydroxylase (F5H) lowered lignin content in the transgenic lines of alfalfa. Repression of C3H provided a better digestibility of alfalfa compared to C4H or F5H (Reddy et al., 2005). Cellulose and hemicellulose are two major components in the plant cell wall. Unlike lignin, cellulose and hemicellulose are partially digestible in rumen. Increasing the amount of either cellulose or hemicellulose is favorable for improvement of alfalfa digestibility (Jung et al., 2012; Adesogan et al., 2019). Several genes, such as glycosyltransferase and beta-glucosidase, have been reported to play important roles on cellulose and hemicellulose biosynthesis and metabolism (Perrin et al., 1999; Scheller and Ulvskov, 2010; Singhania et al., 2013).

Protein content is one of important characteristics affecting the quality of alfalfa. Crude protein (CP) is used for measuring protein in the forage and its content ranges from 18 to 25%. Increasing the intake of protein can significantly improve the cattle growth rate, immunity, and milk production. Acid detergent insoluble crude protein (ADICP) and neutral detergent insoluble crude protein (NDICP) are used to estimate rumen protein availability.

Genetic improvement of forage quality requires understanding the genetic base at the whole genome level. An efficient technique for high-throughput identification of markers/genes responsive to quality traits would be beneficial for genetic improvement. Recently, Next Generation Sequencing (NGS) technology has been used for developing high density SNPs at the genome-wide scale (Rocher et al., 2015). Genome-wide association studies (GWAS) provide advanced tools to identify genetic loci associated with traits of interest using the high-density markers throughout the whole genome (Scheben et al., 2017). It has been successfully applied in the identification of DNA markers associated with agronomic traits in alfalfa and its close relative *M. truncatula* (Alqudah et al., 2020). High-density linkage maps were constructed using GBS markers in both *M. sativa* and *M. truncatula* (Li et al., 2014; Yu et al., 2020). GWAS have been used for identification of significant markers associated with biomass and cell wall biosynthesis in *M. truncatula* (Sakiroglu and Brummer, 2017), and with yield, quality, biotic, and abiotic stress resistance in alfalfa (Biazzi et al., 2017; Hawkins and Yu, 2018; Lin et al., 2020; Medina et al., 2020). However, how genetic factors influence alfalfa quality is still unknown because of the complexity of the alfalfa genome and the quantitative trait controlled by multiple genes.

In this study, a total of 200 alfalfa accessions were planted in three locations for 2 years. Thirty-four quality traits were measured using NIRS. The best linear unbiased estimate (BLUE) was used for analyzing phenotypic variations of each trait. The genotyping was done in the same panel of germplasm by GBS and over 46,000 SNPs were obtained. Marker and trait data were used to identify marker loci associated with quality traits using GWASPoly with multiple models. Our goal is to determine genetic factors that influence forage quality in alfalfa. The genetic information gained from this study would help in genetic improvement of alfalfa with higher quality.

## Materials and Methods

### Plant Materials

A diverse panel of germplasms composed of 148 accessions selected from USDA-ARS National Plant Germplasm System, and 52 cultivars from S&W Seed Co., Alforex Seeds^TM^, Legacy Seeds and Blue River Hybrids were used in this experiment. Hi-Gest360 and 22338_VernalFD2 were used as higher or lower quality checks, respectively. Most of the germplasms were originally collected from Northwest and central regions in US and Canada, including Idaho, Montana, Nebraska, Washington, and North Dakota, South Dakota, British Columbia, Saskatchewan, and Manitoba. Other germplasms were collected from different countries in Asia, Europe, and Africa, including Afghanistan, China, India, Lebanon, Oman, Yemen, Bulgaria, Germany, Russia, Spain, Turkey, and Algeria. All the accessions were listed in Supplementary Table 1.

### Field Experiment

The panel of accessions were planted in three locations in Prosser, Washington (WA), Kimberly, Idaho (ID), and Union, Oregon (OR). Each location contained 11 blocks and each block contains 20 plots with 4.6 m long and 1.2 m wide. An augmented randomized complete block design (ARCBD) was applied in field trial (Federer, 1961; Federer and Raghavarao, 1975). The field at each location was separated to 11 blocks. Twenty accessions including two checks, Hi-Gest^®^, 360 (high quality check) and Vernal (low quality check) were planted in each block. We have one replicate for all accessions but eleven replicates for checks in each location. The phenotypic data from the two checks were used as covariates to correct the phenotypic variation across three locations for 2 years. Field plots were irrigated regularly until harvesting. Samples for quality measurement were collected at mid-bud stage from the first cut in each location. The seeding date in OR, ID, and WA were May 4, May 10, and April 20, respectively, in 2018. The first cut date in OR, ID, and WA were Jul 11, Jul 10, and Jun 29 in 2018, and Jun 4, Jun 5, May 14 in 2019, respectively. The average temperature between seedling date and first cut in 2018 were 15.6, 17.3, and 16.8°C at locations in OR, ID, and WA, respectively. Soil pH varied from 6.3 to 8.2, cation exchange capacity varied from 18.7 to 40.7 across the three locations. Fertilizer nutrients were applied in each location based on the soil parameters.

### Phenotyping and Data Analysis

Shoot samples were collected from first cuts in each location and used for analysis of the 34 traits related to forage quality. The samples collected for quality test were from the same plants for DNA extraction. All samples were dried at 57°C, and then transferred to Hammer mill (Meadow Mills, Wilkesboro, NC 28659) for first grinding and then to Wiley Mill (Thomas Scientific, United States) for second grinding to 2 mm, followed by a final grinding in the sampling mill (Udy Cyclone mill, Fort Collins, CO 80524) through a 1 mm screen. Sample powders were analyzed for 34 quality factors including CP, indigestible CP (ADICP) soluble protein (Protsol), fiber (ADF and NDF), fiber digestibility (NDFD) undigested NDF (uNDF), non-fiber carbohydrates (sugar, NFC) and for several indexes of forage fiber quality (TTNDFD, RFV, and RFQ) and other factors by NIRS using a scanning monochromator (FOSSNIR Systems 5000, Silver Spring, MD, United States) by Rock River Laboratory (Watertown, WI 53094). The reference calibrations for the above components used proprietary equations developed from more than 100,000 alfalfa samples. Global H statistics of each sample were submitted and the average Global H of the sample set was monitored to assure that the spectra of samples submitted were consistent with the calibration samples. Global H is a leverage value scaled so that the average value in the calibration set is 1.0. It measures the squared distance of a spectrum to the average spectrum (Marten et al., 1984; Karn et al., 1991; Shenk and Westerhaus, 1991).

Best linear unbiased estimates (BLUEs) were calculated with the lme4 package in R for analyzing phenotypic data. Genotype was assumed as a fixed effect, and environmental factors such as location, year, and block were treated as a random effect. When analyzing phenotypic data by location, environmental factors included year and block. Analysis of correlation amongst 34 quality traits was performed by Pearson coefficients using BLUEs.

### DNA Samples Preparation and Sequencing

Leaves of 24 representative plants from each accession were collected as a pool for extraction of DNA using Qiagen DNeasy 96 Plant Kit (Qiagen, CA) according to the manufacture’s protocol. DNA samples were tested by measuring the concentration and absorbance ratio of 260/280 and 260/230 with Nanodrop 1000 spectrometer (Thermo Fisher Scientific). DNA samples were sent to the sequencing facility at Oregon State University for library preparation following digestion by restriction enzyme *Ape*KI. Illumina Hiseq2000 system was used for sequencing DNA libraries. To increase coverage, each library was sequenced using two channels with double strands. On average, the sequencing dataset contained over 6 million reads of 136 bp length per sample. The *M. truncatula* genome sequence (Mt A17 r5.0) was used as a reference genome for alignment of the reads using Bowtie2 with highly sensitive parameters (Langmead and Salzberg, 2012). Variant calling was performed using NGSEP v4.0 with the following parameters: maximum base quality score 30, minimum genotyping quality 40, ploidy = 4 (Duitama et al., 2014). The SNPs with minor allele frequency (MAF) lower than 0.05 or missing value higher than 20% were removed. A total of 46,792 (8.1% missing calls) SNPs were obtained after filtering. Missing values in the variants were imputed via hidden Markov model in NGSEP V4.0.

### Population Structure Analysis

The genotypic data was used for principal component analysis (PCA). First, the vcf file containing 46,792 SNPs was transformed to bed format using the plink software. Then eigenvec file containing the data of PC1, PC2, and PC3 was generated using GCTA (Yang et al., 2011). A phylogenetic tree was generated using the neighbor joining method and plotted by iTOL (Ciccarelli et al., 2006).

### Genome-Wide Association Analysis and Identification of Candidate Genes

The R package of GWASpoly, which takes the allele dosage into account, was used for GWAS (Rosyara et al., 2016). Six different marker-effect models including additive, general, diplo-general, diplo-additive, 1-dom and 2-dom were applied for analysis of marker-trait association. Both Q and K matrix were included in the analysis. Q matrix was obtained from PCA. K matrix was determined by default in GWASpoly after inputting the phenotype and SNP data. False discovery rate (FDR) of 0.05 was used as threshold for significance selection. Candidate genes were localized by retrieving the positions of significant markers in *M. truncatula* genome database^1^.

### Gene Annotation

Gene annotation was based on the SNP position in *M. truncatula* genome dataset V5.0. National Center for Biotechnology Information^2^ and UniProt database^3^ were also used for annotation by extracting a 2-kb flanking region of each significant marker and searching the homologs by BLAST.

## Results

### Development of SNP Markers Using Genotyping by Sequencing

**FIGURE 1:**
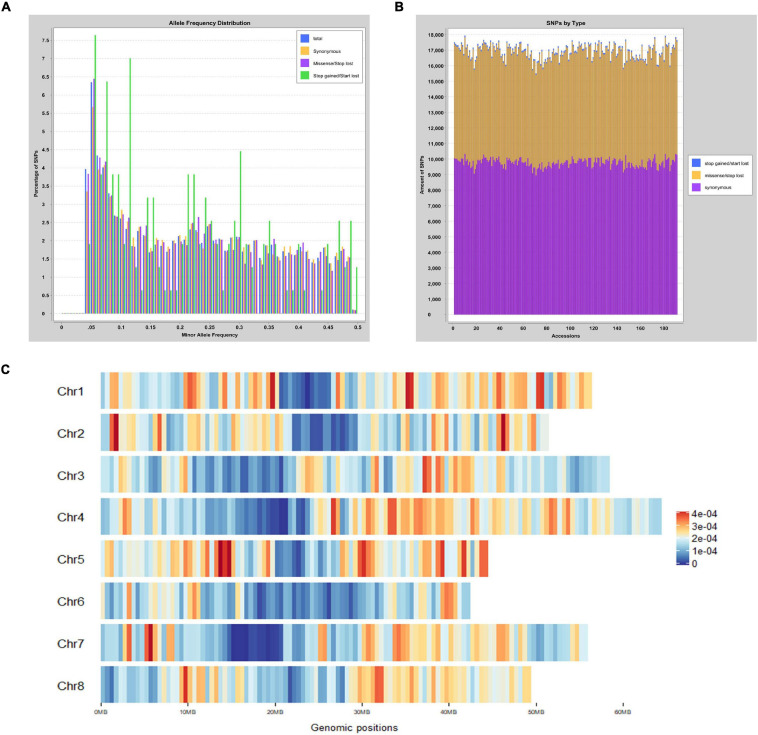
Distribution of MAF and SNP across the *Medicago truncatula* genome. **(A)** Distribution of MAF from 0.05 to 0.5. Different types of variants were displayed in three colors. Synonymous variants are shown in yellow, whereas missense/stop lost variants and stop gained/start lost variants are shown in purple and green, respectively. **(B)** Statistics of three different types variants. Each accession is presented in a single vertical bar. The proportion of variants including synonymous, missense/stop lost, and stop gained/start lost were shown in purple, yellow, and blue, respectively. **(C)** SNP distribution and density on each chromosome. Loci with high density SNPs were shown in red color, whereas low density SNPs were shown as blue. The marker at the bottom displays the lengths of each chromosome and the positions of SNPs.

GBS generated more than 1,146 million reads with the average length of 136 bases. More than 53.9% reads were successfully aligned to the *M. truncatula* reference genome. Minor allele frequency (MAF) was distributed between 0.05 and 0.5 (Figure 1A). The proportion of SNPs with MAF between 0.05–0.1, 0.1–0.2, 0.2–0.3, 0.3–0.4, and 0.4–0.5 were 24.7, 20.3, 21.0, 18.0, and 16.1%, respectively. After variant calling and filtering, 46,792 meaningful SNPs were obtained. Among those, 4,796 were intergenic variants and up to 63.8% SNPs were present in the coding regions with 13,040 synonymous variants and 9,796 missense variants (Figure 1B). Six hundred and twenty-three and 1,955 variants were identified at the 5′ and 3′ untranslated regions (UTR), respectively. In addition, there were 923 variants located at the upstream of the 5′ UTR, while 419 variants were in the downstream region. The distribution and density of the SNPs on 8 chromosomes were illustrated in Figure 1C. Chromosome 3 had the most with 7,258 SNPs. The highest density of SNPs was observed on chromosome 1 with 122.98 SNPs in every mega-base pair (Table 1).

**TABLE 1:** SNP distribution and frequency across the genome of *M. truncatula*.

**Chromosome**	**Number of SNPs**	**Lengths (bp)**	**SNPs/Mb**
1	6,974	56,706,830	122.98
2	5,741	51,972,579	110.46
3	6,037	58,931,556	102.44
4	7,258	64,763,011	112.07
5	5,760	44,819,618	128.52
6	3,549	42,866,092	82.79
7	5,745	56,236,587	102.16
8	5,627	49,719,271	113.18
0c01	18	1,205,179	14.94
0c05	17	150,389	113.04
0c09	5	63,286	79.01
MT	61	271,618	224.58

**0c01, 0c05, and 0c09 are scaffolds; MT, mitochondria.**

### Statistical Analysis of Quality Traits

**FIGURE 2:**
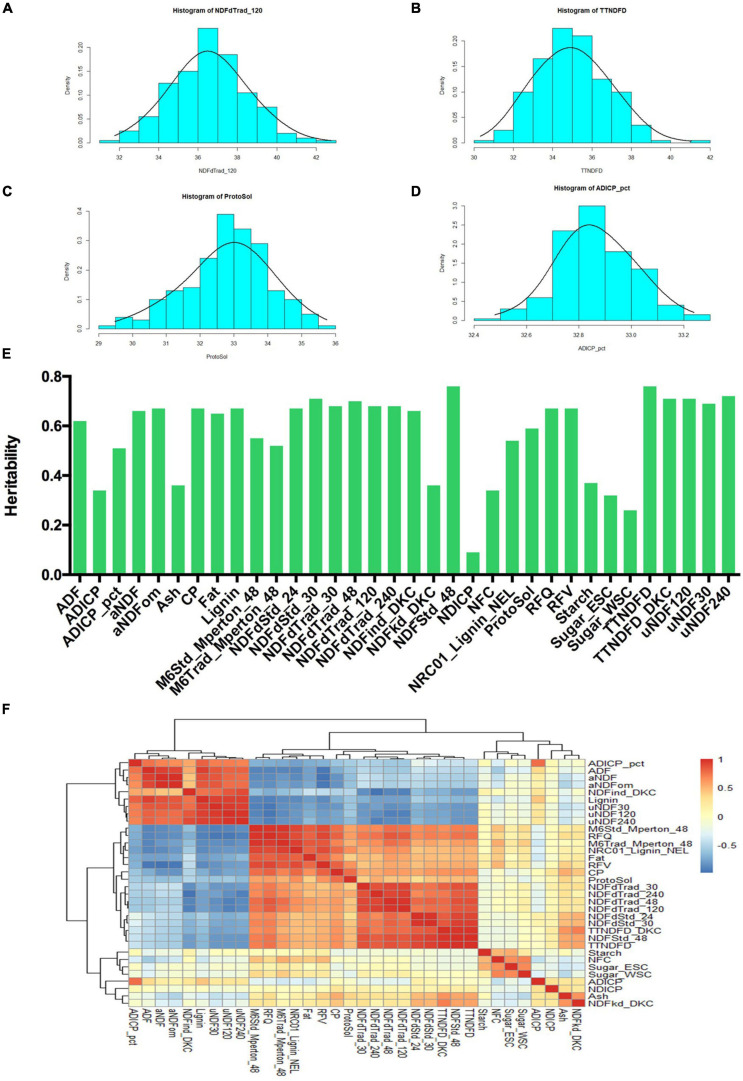
Analysis of phenotypic variance in quality traits of alfalfa accessions. Histogram showed the phenotypic value of NDFdTrad_120 **(A)**, TTNDFD **(B)**, ProtoSol **(C),** and ADICP_pct **(D)**. **(E)** Broad sense heritability of 34 quality traits. All the 34 quality traits are listed on X-axis, and heritability value is shown on Y-axis. **(F)** Correlation analysis of 34 quality traits based on Pearson coefficients. Positive correlations were displayed in red, whereas negative correlations were showed in blue. All the 34 quality traits were hierarchically clustered as shown on the top and left side of the figure.

Statistical analyses were performed among all 34 quality traits. Frequency distributions of 4 quality traits (NDFdTrad_120, TTNDFD, ProtoSol, ADICP_pct) related to fiber digestibility and protein content were shown in Figure 2. The phenotypic data of all the traits were normally distributed. NDFdTrad_120 varied from 57.97 to 69% (Figure 2A). The minimum and maximum values of TTNDFD were 40.23 and 50.97%, respectively (Figure 2B). ProtoSol (soluble protein) was ranging from 32.43 to 38.71%, with the mean value of 35.78% (Figure 2C). ADICP_pct varied from 2.12 to 2.89%, with the mean value of 2.51% (Figure 2D). Other traits were also well distributed as shown in histograms (Supplementary Figure 2). Broad sense heritability (H^2^) was calculated for all 34 traits (Figure 2E). The H^2^ of 26 traits were greater than 0.5. Among them, the H^2^ values of 6 traits (NDFStd_48, TTNDFD, uNDF240, uNDF120, TTNDFD_DKC, and NDFdStd_30) were higher than 0.7, indicating most of phenotypic variations of these traits were genetically controlled. Lower heritability was found in Sugar_WSC and NDICP with H^2^ less than 0.3, with the lowest heritability of 0.1 in NDICP. The correlations between 34 traits were analyzed based on Pearson coefficients and 3 groups were classified (Figure 2F). Among the highly positive correlation groups, TTNDFD is highly correlated to NDFStd_48, with the coefficient value of 0.99. The coefficient between aNDF and aNDFom was 0.98, followed by TTNDFD_DKC and TTNDFD, NDFdTrad_48, and NDFdTrad_120, with the coefficients greater than 0.97. High correlations were also detected between RFQ and M6Std_Mperton_48, NDFdStd_24 and NDFdStd_30, M6Trad_Mperton_48 and M6Std_Mperton_48, NDFStd_48 and TTNDFD_DKC, uNDF30 and uNDF120, and uNDF240 and uNDF30, with the coefficients higher than 0.95. In addition, a highly negative correlation (−0.99) was observed between NDFind_DKC and NDFdTrad_240. RFV was negatively correlated with ADF, aNDF, and aNDFom, with coefficients lower than −0.96. RFQ was also negatively correlated with uNDF120 and uNDF30, with the coefficients of −0.96 for both. Starch, NFC, Sugar_ESC, Sugar_WSC, ADICP, NDICP, Ash, and NDFkd_DKC were classified into the intermediate group according to their correlations. Moderately positive correlation was observed between ADICP and NDFkd_DKC with the coefficient 0.25, whereas Sugar_ESC and NDFkd_DKC were in moderately negative correlation with the coefficient of −0.35.

### Population Structure Analysis

**FIGURE 3:**
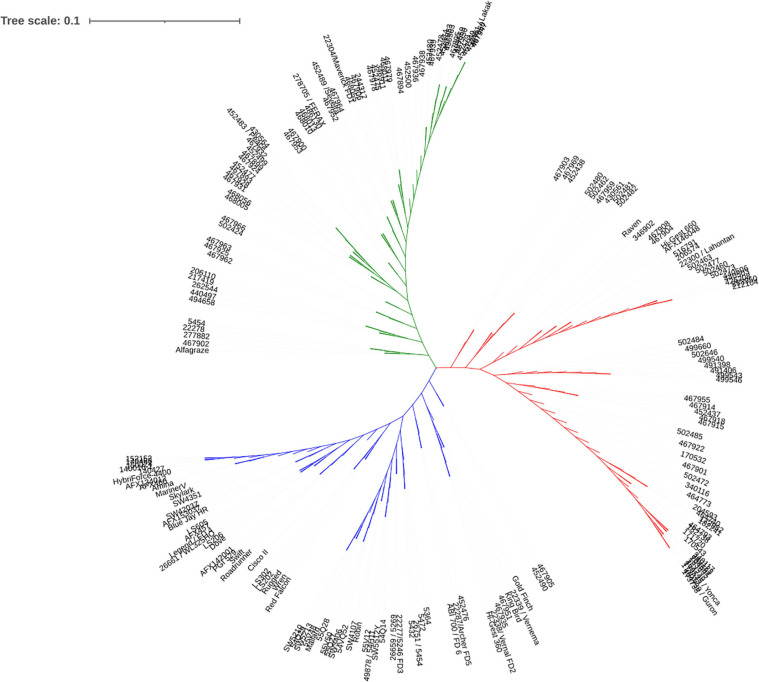
Population structure based on phylogenetic analysis. Clusters 1, 2, and 3 were displayed in green, red, and blue, respectively.

Genotypic data were used for cluster analysis using neighbor joining method. The alfalfa accessions were grouped into 3 clusters (Figure 3). Among those, 63 accessions were classified to cluster 1, 64 accessions were classified to cluster 2 and 65 accessions were in cluster 3. Cluster 1 contained a mixture of accessions from multiple countries and regions, including United States, Canada, China, Mediterranean, and central and eastern Europe. Cluster 2 contained the accessions from Turkey and central Asia. Cluster 3 contained accessions from United States except one from Canada. The result was consistent with the PCA where similar groups were observed (Supplementary Figure 1).

### Genome-Wide Association Analysis

GWASPoly was used for GWAS with allelic dosage using different models, and the FDR of 0.05 was used as the threshold for the selection of significant associations. In total, 28 significant markers were obtained. Several markers were identified in more than one model. Generally, the traits associated with these 28 markers can be categorized into two groups.

**FIGURE 4:**
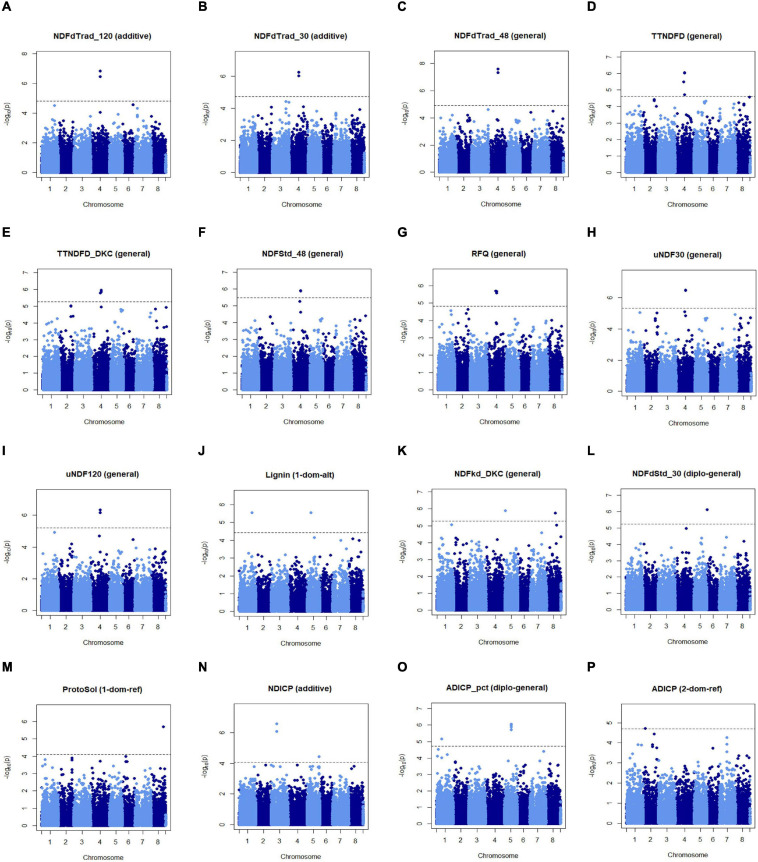
Manhattan plots of marker-trait association of alfalfa quality traits. Significant markers were identified in multiple traits including NDFdTrad_120 **(A)**, NDFdTrad_30 **(B)**, NDFdTrad_48 **(C)**, TTNDFD **(D)**, TTNDFD_DKC **(E)**, NDFStd_48 **(F)**, RFQ **(G)**, uNDF30 **(H)**, uNDF120 **(I)**, Lignin **(J)**, NDFkd_DKC **(K)**, NDFdStd_30 **(L)**, ProtoSol **(M)**, NDICP **(N)**, ADICP_pct **(O),** and ADICP **(P)**. Chromosome positions were based on the genome of *M. truncatula* v5.0. X-axis shows the chromosome numbers, and Y-axis shows the -log *p*-value.

The first group contained markers associated with traits related to fiber digestibility, including Lignin, uNDF30, uNDF120, TTNDFD, TTNDFD_DKC, NDFdTrad_30, NDFdTrad_48, NDFdTrad_120, NDFdStd_30, NDFStd_48, and RFQ. Two markers, MtChr4_33424222, and MtChr4_33424238 (at the same locus) on chromosome 4 were associated with 9 traits related to fiber digestibility, including NDFdTrad_120, NDFdTrad_30, NDFdTrad_48, TTNDFD, TTNDFD_DKC, NDFStd_48, RFQ, and uNDF30 as well as uNDF120 (Figures 4A–I). Additionally, three markers including two on chromosome 2 (MtChr2_45854840, MtChr2_46050634) and one on chromosome 8 (MtChr8_47585557) were associated with NDFdTrad_30 (Supplementary Figure 3). Markers associated with Lignin content were identified on chromosome 1 (MtChr1_43382331) and chromosome 5 (MtChr5_13533783) (Figure 4J). Moreover, the marker MtChr1_43382331 was also associated with uNDF30 and uNDF120 (Supplementary Figure 3). Marker MtChr4_30582636 identified on chromosome 4 was associated with TTNDFD, TTNDFD_DKC, and RFQ (Figures 4D,E,G). Another TTNDFD-associated marker MtChr4_32514187 was identified on chromosome 4 (Figure 4D). MtChr1_48744767 and MtChr6_522150 were associated with TTNDFD_DKC (Supplementary Figure 3). Two markers MtChr5_1952458 and MtChr8_30029709, associated with NDFkd_DKC, were identified on chromosomes 5 and 8, respectively (Figure 4K). Marker MtChr6_1927418 associated with NDFdStd_30 with -log *p*-value 6.11, was detected on chromosome 6 (Figure 4L and Supplementary Figure 3).

The second group contained markers associated with traits related to protein content, including ProtoSol, ADICP, ADICP_pct, and NDICP. Both ADICP and NDICP are measurements of insolubility of crude protein. A significant marker on chromosomes 8 (MtChr8_41724151) was associated with ProtoSol (Figure 4M). MtChr3_20251386 and MtChr3_20251391 on chromosome 3 with MtChr5_41544991 on chromosome 5 were associated with NDICP (Figure 4N). Seven markers significantly associated with ADICP_pct were on chromosome 5 and detected in different models (Figure 4O and Supplementary Figure 3). Besides the 7 clustered SNPs, another two markers MtChr1_16144476 and MtChr1_36174006 on chromosome 1 were also associated with ADICP_pct. An ADICP associated marker MtChr2_6808038 was detected on chromosome 2 (Figure 4P).

### Annotation of Functional Genes Associated With Quality Traits

The flanking sequences of significant markers were used for searching putative candidate genes using the BLAST search as described above. Of the 28 significant markers identified, 27 were encompassed by the annotated genes with different functions (Table 2). ProtoSol-associated marker MtChr8_41724151 was in a gene region of methyltransferase. Markers (MtChr1_16144476 and MtChr1_36174006) associated with ADICP_pct on chromosome 1 were adjacent to genes encoding Casein kinase 1 (CK1) family protein kinase and broad complex, tram track, bric-a-brac/POX virus and zinc finger (BTB/POZ) protein, respectively. Marker MtChr2_6808038 associated with ADICP was closely located to the gene encoding a tubby-like protein. Two of the NDFdTrad_30-associated markers on chromosomes 2 and 8 were closely located at the genes encoding thioredoxin-like protein and E3 ubiquitin-protein ligase, respectively. The TTNDFD-associated marker MtChr4_32514187 was located at the coding region of sulfurtransferase gene. Two markers, MtChr3_20251386 and MtChr3_20251391, were located at the same locus and they were associated with NDICP. They were encompassed by the gene encoding 1,3-beta-glucan synthase. Another NDICP associated gene surrounding MtChr5_41544991 was annotated as U3 small nucleolar RNA-associated protein. MtChr4_30582636 was in the coding region of a gene homologous to 2′,3′-cyclic-nucleotide 3′-phosphodiesterase. Three markers MtChr5_1952458, MtChr8_30029709, and MtChr_43382331, associated with fiber related traits, were annotated as cycloartenol synthase, 1-phosphatidylinositol 4-kinase, and sodium/calcium exchanger membrane protein, respectively.

**TABLE 2:** Significant markers associated with quality traits identified using *M. truncatula* genome as references.

**Marker**	**Ref**	**Alt**	**-log *p***	***R*^2^**	**Trait**	**Annotation**
MtChr8_41724151	A	G	5.7	0.2	ProtoSol	Putative tRNA [cytosine(34)-C(5)]-methyltransferase
MtChr2_6808038	A	G	4.71	0.06	ADICP	Putative transcription factor TUBBY family
MtChr1_16144476	A	G	5.15	0.19	ADICP_pct	Putative protein kinase CK1-CK1 family
MtChr1_36174006	A	G	5.38	0.27		Chromatin remodeling and transcription regulator BTB-POZ-MATH family
MtChr5_24576491	C	G	5.72	0.09		Hypothetical protein
MtChr5_24576492	A	T	6.04	0.09		
MtChr5_24576497	C	T	6.01	0.1		
MtChr5_24576498	G	T	6.04	0.09		
MtChr5_24576499	A	T	5.9	0.09		
MtChr5_24576500	A	G	6.04	0.09		
MtChr5_24576507	A	T	6.07	0.1		
MtChr3_20251386	C	T	6.56	0.12	NDICP	1,3-Beta-glucan synthase
MtChr3_20251391	A	G	6.06	0.11	NDICP	
MtChr5_41544991	A	G	4.43	0.08	NDICP	U3 small nucleolar RNA-associated protein
MtChr1_43382331	A	T	5.55	0.16	Lignin	Sodium/calcium exchanger membrane region
	A	T	5.73	0.18	uNDF120	
	A	T	5.82	0.18	uNDF30	
MtChr2_45854840	A	C	5.29	0.01	NDFdTrad_30	Hypothetical protein
MtChr2_46050634	A	G	5.34	0.01	NDFdTrad_30	Putative thioredoxin-like protein
MtChr8_47585557	C	T	5.5	0.01	NDFdTrad_30	Aminoacyltransferase, E1 ubiquitin-activating enzyme
MtChr4_30582636	C	T	5.7	0.09	RFQ	2′,3′-cyclic-nucleotide 3′-phosphodiesterase
	C	T	5.49	0.04	TTNDFD	
	C	T	5.79	0.04	TTNDFD_DKC	
MtChr4_32514187	A	C	4.71	0.03	TTNDFD	Sulfurtransferase
MtChr4_33424222	C	T	5.62	0.21	NDFdTrad_120	Hypothetical protein
	C	T	6.25	0.19	NDFdTrad_30	
	C	T	7.59	0.21	NDFdTrad_48	
	C	T	5.92	0.15	NDFStd_48	
	C	T	5.68	0.16	RFQ	
	C	T	6.06	0.15	TTNDFD	
	C	T	5.86	0.14	TTNDFD_DKC	
	C	T	6.33	0.19	uNDF120	
	C	T	6.48	0.17	uNDF30	
MtChr4_33424238	C	T	5.26	0.2	NDFdTrad_120	
	C	T	6.02	0.19	NDFdTrad_30	
	C	T	7.32	0.21	NDFdTrad_48	
	C	T	5.88	0.15	NDFStd_48	
	C	T	5.59	0.16	RFQ	
	C	T	6.03	0.16	TTNDFD	
	C	T	5.94	0.15	TTNDFD_DKC	
	C	T	6.16	0.18	uNDF120	
	C	T	6.47	0.17	uNDF30	
MtChr5_13533783	A	G	5.55	0.11	Lignin	Hypothetical protein
MtChr5_1952458	G	T	5.9	0.01	NDFkd_DKC	Cycloartenol synthase
MtChr8_30029709	A	G	5.75	0.07	NDFkd_DKC	1-Phosphatidylinositol 4-kinase
MtChr6_1927418	A	G	6.11	0.04	NDFdStd_30	Preprotein translocase subunit SecE
MtChr1_48744767	G	T	4.6	0.21	TTNDFD_DKC	Hypothetical protein
MtChr6_522150	C	T	4.56	0.11	TTNDFD_DKC	840 bp away from a hypothetical gene

Additionally, MtChr4_33424222 and MtChr4_33424238, associated with multiple fiber related traits, were located near a hypothetical protein with unknown function. Similarly, markers MtChr5_13533783 and MtChr2_45854840 associated with Lignin and NDFdTrad_30, respectively, were also located at the gene regions of hypothetical proteins, as well as a cluster of markers associated with ADICP_pct on chromosome 5.

### Comparison of Markers Identified in Different Environments

**FIGURE 5:**
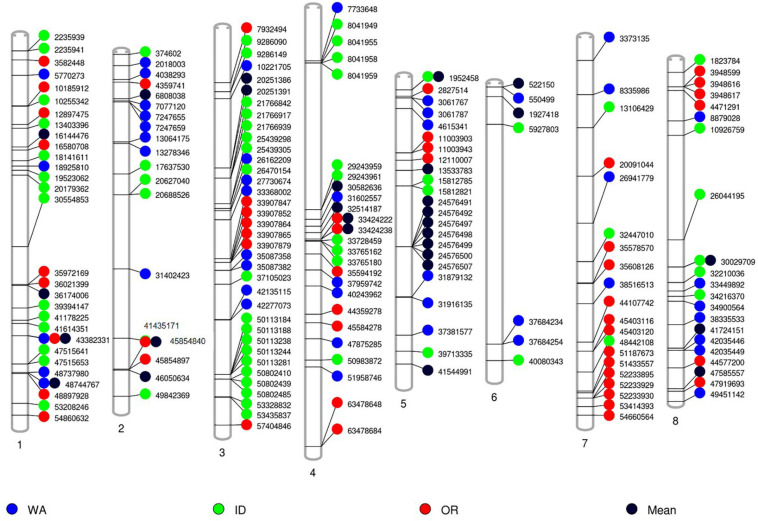
Identification of significant markers associated with alfalfa quality using phenotypic data by location. Markers identified in Washington (WA), Idaho (ID), and Oregon (OR) were displayed in blue, green, and red, respectively. Black spots indicate significant markers based on adjusted means of all quality data collected from three locations for 2 years.

To investigate markers associated with alfalfa quality traits under different environments, we used the means of 2-year phenotypic data collected from different locations and analyzed separately by location with the same genotypic data for GWAS. The average value of CP content in WA was lower than those in ID and OR, while ProtoSol was the highest in OR among the 3 locations. For digestibility-related traits such as NDFdTrad_120 and TTNDFD, average values were higher in ID than those in OR and WA. However, WA had the highest values of Lignin and uNDF traits. The phenotypic variations of accessions of 34 quality traits by location are presented in Supplementary Figure 4. One hundred and fifty-six markers were associated with 24 quality traits (Figure 5). 47, 64, and 46 markers were identified in WA, OR, and ID, respectively (Figure 5 and Supplementary Tables 2–4). Compared with those identified using adjusted means from three locations, two, two, and four markers from WA, ID, and OR, respectively, overlapped with those identified using adjusted means. One marker (MtChr1_43382331) associated with Lignin was identified in WA, OR, and the adjusted means. This marker was associated with TTNDFD and NDFStd_48 that negatively correlated with Lignin. The rest of markers were not overlapping with those identified using adjusted means, indicating alfalfa quality was also strongly affected by environments. Markers consistently expressed in different locations will be useful in MAS after validation.

## Discussion

### Genotyping Call and GWAS With Allelic Dosage

In this study, we used GBS for genotyping the association panel of 200 alfalfa varieties and obtained over 46,000 SNPs. These high-density markers were subsequently used for association mapping of 34 alfalfa quality traits in the panel of association. Although several gaps were observed on chromosomes, most of these regions were potentially close to centromere that are considered to contain redundant repeats and methylated sequences with lower gene density (Wong et al., 2006; Mizuno et al., 2011).

Genetic studies in tetraploid species such as alfalfa and potato have lagged behind those in diploids because segregation patterns are more complex. Most of the software are designed for diploid species (Bourke et al., 2018). Until recently, software such as GWASPloy has been developed for GWAS in polyploid species. GWASPoly takes allele dosage into account for a given genetic locus with multiple alleles (e.g., aaaa, baaa, bbaa, bbba, and bbbb). The use of GWASPoly in the present analysis identified 28 loci associated with forage quality with multiple alleles and the regression analysis highlighted correlations between phenotypes and allelic genotypes. For instance, marker Chr8_41724151 associated with ProtoSol showed heterozygous alleles of AAAG, AAGG, and AGGG among the top 20 entries with high ProtoSol, whereas the bottom 20 entries on ProtoSol had homozygous alleles of GGGG at the same locus (Supplementary Table 5). Homozygous allele GGGG at locus Chr1_36174006 was observed in 75% entries with high ADICP_pct, whereas the bottom 20 entries with low ADICP_pct had heterozygous allele GGGA or GGAA. For NDFD, entries with lowest values of NDFdTrad_48, showed TTTT at marker locus Chr4_33424222, while 60% genotypes with higher NDFdTrad_48 values had TTTC or TTCC alleles. High correlations between alleles and phenotypes at loci associated with important quality traits such as protein content and digestibility provide more clear genetic profiles for the traits.

### Correlations Between Fiber- and Energy-Related Traits

Alfalfa quality is determined by a variety of parameters. The measurement of 34 quality-related parameters in this study almost covered all the essential quality-related characteristics. Generally, high quality forage is rich in protein and can supply high amount of nutrients to the livestock (Hoveland and Monson, 1980). However, crude protein content and the digestion rate decline during the maturity of alfalfa. Analysis of the correlation between protein content and digestibility would provide a better clue on the quality changes during the growth period of alfalfa. In the present study, the result of the correlation analysis found a total of 97 pairs of traits that were strongly positively correlated with the coefficients greater than 0.7, whereas other pairs of traits were strongly negatively correlated with the coefficients lower than −0.7. The positive correlations were mainly observed in two groups of traits. The first group included ADF, aNDF, aNDFom, ADICP_pct, NDFind_DKC, Lignin, uNDF30, uNDF120, and uNDF240. The second group included M6Std_Mperton_48, M6Trad_Mperton_48, NRC01_Lignin_NEL, Fat, RFV, RFQ, CP, ProtoSol, NDFdTrad_30, NDFdTrad_48, NDFdTrad_120, NDFdTrad _240, NDFdStd_24, NDFdStd_30, NDFStd_48, TTNDFD, and TTNDFD_DKC. It was not surprising that the traits within the groups were positively correlated as they are in the same categories. Likewise, negative correlations were found between the two groups as they contained opposite traits. Although some traits are highly correlated, the genetic basis behind each trait may be different. Moreover, each of the traits has different characteristics when evaluating alfalfa quality. For example, Lignin, ADF, and NDF are highly correlated as both lignin and cellulose are counted in ADF and hemicellulose is counted in NDF besides lignin and cellulose. NDFD240 represents the indigestible fiber and it is related to gut fill in ruminants (Cotanch, 2015). NDFD30 and NDFD48 are usually used to index how fast fiber degrades (Hoffman and Combs, 2003). On the other hand, the result of correlations among those traits are also helpful to simplify the evaluation process on alfalfa quality in the future since it tells us which traits are highly correlated.

### Markers Associated With Multiple Highly Correlated Traits

In present study, we identified two markers, MtChr4_33424222 and MtChr4_33424238, at the same locus associated with nine traits. All of them were related to fiber digestibility and they were highly correlated with each other. The highest correlation was found between NDFStd_48 and TTNDFD with the coefficient value of 0.99, whereas the lowest coefficient 0.69 was observed between RFQ and TTNDFD_DKC among the nine traits. The most negatively correlated pair was uNDF120 and RFQ with coefficient −0.96. In general, uNDF120 and uNDF30 were negatively correlated with the other seven traits including NDFdTrad_30, NDFdTrad_48, NDFdTrad_120, NDFStd_48 RFQ, TTNDFD, and TTNDFD_DKC, as uNDF120 and uNDF30 reflected the amount of undigested fiber which are negative factors for alfalfa quality. The associations between these two markers and the nine fiber-related traits indicate that the associated loci are possibly involved in the regulation of multiple pathways including lignin biosynthesis and other cell wall components such as cellulose or hemicellulose. Further investigation of the candidate genes underlying these loci would be helpful to understand the genetic basis of fiber digestibility in alfalfa.

### Putative Candidate Genes With Functions Involved in Forage Quality

Among significant markers identified in the present study, fourteen were associated with fiber-related traits; The same significant markers, MtChr4_33424222 and MtChr4_33424238 were detected in nine fiber-related quality traits and they were highly correlated each other. In our previous study, over 100 markers were associated with alfalfa quality under drought conditions (Lin et al., 2020). Our present results showed multiple candidate genes associated with forage quality which had similar functions to genes identified in the previous study. Sakiroglu and Brummer (2017) found that a gene locus associated with ADF and NDF on chromosome 8 was involved in cell wall biosynthesis, arabinose, and xylose content. Biazzi et al. (2017) identified three markers associated with CP, NDF digestibility (NDFD), and acid detergent lignin (ADL) of leaf, together with multiple significant markers in association with CP and ADL of stem. We identified 28 significant markers associated with protein content and fiber-related traits. Alfalfa with low content of lignin is highly desirable as rumen digestibility is negatively correlated with lignin content of alfalfa (Reddy et al., 2005). In the present study, marker MtChr1_43382331 was annotated as a sodium/calcium exchanger. This locus was associated with both uNDF120 and lignin, indicating that the exchange of sodium and calcium is likely to affect the composition of cell wall. The calcium level in plants is in a strong relationship with the amount of pectin in then cell wall (Reid and Smith, 2000). Furthermore, calcium homeostasis and binding on membranes of plant cells is affected by sodium (Rengel, 1992). Marker MtChr5_1952458 is associated with NDFkd_DKC, located in the gene coding region of cycloartenol synthase. It has been reported that cellulose biosynthesis is strikingly influenced by the sterol composition, and the high cycloartenol content often causes a disrupted cell wall structure and reduced cellulose (Schrick et al., 2004). Marker MtChr2_46050634 associated with NDFdTrad_30 was located at the region of gene encoding thioredoxin-like protein. Thioredoxin is a class of protein involving redox signaling and relief of oxidative stress. It exists in a variety of organisms and is involved in many biological processes (Gelhaye et al., 2005). Recent studies showed that thioredoxin-like protein affects lignin deposition, polymerization and cell wall synthesis (Alkhalfioui et al., 2007; Shin et al., 2020). Another marker (MtChr8_30029709) associated with NDFkd_DKC was located at the gene region of 1-phosphatidylinositol 4-kinase (PIK4), a transferase responsible for transferring phosphorus-containing groups. A recent study showed that PIK4 plays an important role in the trafficking of cellulose synthase complexes (Fujimoto et al., 2015). Marker MtChr4_30582636 was in the gene encoding region of 2′,3′-cyclic-nucleotide 3′-phosphodiesterase (CNPase), a member of the 2H phosphoesterase superfamily. This type of enzyme has RNA ligase activity and may also be involved in tRNA splicing in plants. Slabas et al. (2004) detected t-RNA ligase in cell wall of Arabidopsis, implying the potential function of the enzyme on cell wall synthesis. Marker MtChr8_47585557 in candidate gene encoding E3 ubiquitin ligase was also associated with NDFdTrad_30 in the present study. E3 ubiquitin ligase is a protein that recruits ubiquitin and catalyzes its transfer from E2 subunit to the substrate directly or indirectly (Buetow and Huang, 2016). Borah and Khurana (2018) found that E3 ligase subunit F-box Kelch 1 (FBK1) was involved in root secondary cell wall thickening and lignin biosynthesis in rice. Another ubiquitin ligase protein, Arabidopsis Tóxicosen Levadura54 (ATL54) identified in Arabidopsis, participated in programmed cell death and secondary cell wall formation, although no significant variance on lignin content and composition in whole mature stem was observed by altering the expression level of *ATL54* (Noda et al., 2013). TTNDFD associated marker MtChr4_32514187 was encompassed by a gene encoding sulfurtransferase (Str). The interaction between Str and thioredoxin was found in different organisms, suggesting the involvement of Str in redox homeostasis, in turn affecting lignin biosynthesis (Papenbrock et al., 2011).

ADICP associated marker MtChr2_6808038 was in a *Tubby* gene. Tubby-like protein (TLP) is a large family and widespread in many plant species. However, only a small proportion of TLPs referring to stress response and pollen development have been identified in plants (Wang et al., 2018). In the present study, marker MtChr2_6808038 identified in the gene region of TLP was associated with ADICP, which represents the portion of indigestible protein in rumen. The same gene was also identified previously in association with dry matter yield and early seedling growth (Sakiroglu and Brummer, 2017). Two annotated genes closely located with three markers were associated with NDICP, which is fiber-bound and considered to be an indigestible protein in rumen and the small intestine. One of the genes encoding 1,3-beta-glucan synthase is responsible for callose formation in plants. Callose is a polysaccharide in plant cell walls and important for a variety of processes in plant development and response to biotic and abiotic stresses (Chen and Kim, 2009). In addition, Cell wall rigidity was changed by regulating expression of this type of gene in Arabidopsis (Oliveira-Garcia and Deising, 2013).

To explore more candidate genes, we extended the search to the 50-kb region surrounding the significant SNPs (Table 3). Several genes were found to be potential candidates based on the gene functions. For example, a gene encoding pectinesterase was close to MtChr1_43382331, which catalyses the de-esterification to release pectin and methanol (Micheli, 2001). MtChr4_33424222 and MtChr4_33424238 were associated with nine quality traits, all of which were NDF-related traits with strong correlations each other. These two markers were adjacent to a beta-glucosidase gene less than 30 kb. Beta-glucosidase is an essential enzyme responsible for the hydrolysis of lignocellulose and plays important roles in cell wall formation (Singhania et al., 2013; Tsuyama and Takabe, 2015; Sampedro et al., 2017). NDFD associated marker MtChr6_1927418 was around 20 kb away from a gene encoding glycosyltransferase, which is implicated in the synthesis of polysaccharide backbone and side-chain linkages in the cell wall (Amos and Mohnen, 2019). Two genes, MtA17Chr6g0449561 and MtA17Chr6g0449521, encoding galactose oxidase and galactinol-sucrose galactosyltransferase were downstream and upstream of marker MtChr6_522150, respectively. Galactose oxidase is related to pectin synthesis and influences pectin properties (Šola et al., 2019), and involved in the modification and degradation of lignocellulose (Marjamaa and Kruus, 2018). Galactinol-sucrose galactosyltransferase belongs to glycosyltransferase, which is also involved in cell wall formation.

**TABLE 3:** Potential candidate genes within 50-kb of significant SNPs.

**Trait**	**SNP**	**Adjacent gene**	**Annotation**	**Range**
Lignin	MtChr1_43382331	MtrunA17Chr1g0195561	Pectinesterase	43,434,578–43,435,483
TTNDFD_DKC	MtChr1_48744767	MtrunA17Chr1g0202641	Galacturan 1,4-alpha-galacturonidase	48,714,585–48,719,814
NDFdTrad_120	MtChr4_33424222	MtrunA17Chr4g0033401	Beta-glucosidase	33,394,821–33,397,755
TTNDFD	MtChr4_32514187	MtrunA17Chr4g0032331	Hexosyltransferase	32,456,246–32,458,704
Lignin	MtChr5_13533783	MtrunA17Chr5g0412101	7-Deoxyloganetic acid glucosyltransferase	13,557,474–13,559,854
NDFdStd_30	MtChr6_1927418	MtrunA17Chr6g0451381	Glycosyltransferase	1,948,594–1,953,305
TTNDFD_DKC	MtChr6_522150	MtrunA17Chr6g0449561; MtrunA17Chr6g0449521	Galactose oxidase; galactinol—sucrose galactosyltransferase	537,765–539,858; 493,534–498,577

In summary, using GBS and GWAS, we developed 46,792 high quality SNPs and identified 28 significant markers associated with 16 quality-traits. Notably, some of the markers, such as MtChr4_33424222 and MtChr4_33424238 were associated with nine traits. Another lignin associated marker MtChr1_43382331 was consistently identified in multiple environments. Putative candidate genes underlying associated loci were identified. They may play roles in regulating forage quality. These markers and genes are going to be validated in our future studies. Once validated, they can be used for developing alfalfa with improved quality. Additionally, allelic dosage helped in finding high correlations between phenotypic traits and alleles at the associated loci and deepened our understanding of how genetic factors influence forage quality.

## Data Availability Statement

The original contributions presented in the study are publicly available. This data can be found here: Sequence Read Archive (SRA) under accession PRJNA666630.

## Author Contributions

L-XY and ON conceived the study. SL and CM carried out the bioinformatic analysis. SL and L-XY wrote the manuscript. ON, DC, GW, GS, SF, and DL conducted field trails and collected samples for quality evaluation. All authors contributed to the article and approved the submitted version.

## Conflict of Interest

The authors declare that the research was conducted in the absence of any commercial or financial relationships that could be construed as a potential conflict of interest.

## Footnotes

1 https://medicago.toulouse.inra.fr/MtrunA17r5.0-ANR/

2 https://www.ncbi.nlm.nih.gov/

3 https://ebi12.uniprot.org/

## Supplementary Material

Supplementary Figure 1 Population structure with principal component analysis. The top 2 components were used to represent the structure. Clusters 1, 2, and 3 were displayed in green, red and blue respectively.

Supplementary Figure 2 Frequency distribution of different quality traits in alfalfa. **(A)** ADF, **(B)** ADICP, **(C)** aNDF, **(D)** aNDFom, **(E)** Ash, **(F)** CP, **(G)** Fat, **(H)** Lignin, **(I)** M6Std_Mperton_48, **(J)** M6Trad_Mperton_48, **(K)** NDFdStd_24, **(L)** NDFdStd_30, **(M)** NDFdTrad_30, **(N)** NDFdTrad_48, **(O)** NDFdTrad_240, **(P)** NDFind_DKC, **(Q)** NDFkd_DKC, **(R)** NDFStd_48, **(S)** NDICP, **(T)** NFC, **(U)** NRC01_Lignin_NEL, **(V)** Sugar_ESC, **(W)** RFQ, **(X)** RFV, **(Y)** Starch, **(Z)** Sugar_WSC, **(AA)** TTNDFD_DKC, **(AB)** uNDF30, **(AC)** uNDF120, **(AD)** uNDF240.

Supplementary Figure 3 Manhattan plots of significant markers associated with alfalfa quality traits by additional models.

Supplementary Figure 4 Phenotype distribution of 34 traits in 3 locations.

Supplementary Table 1 Alfalfa accessions used in phenotype analysis and GWAS.

Supplementary Table 2 Markers identified using phenotype data collected in WA.

Supplementary Table 3 Markers identified using phenotype data collected in ID.

Supplementary Table 4 Markers identified using phenotype data collected in OR.

Supplementary Table 5 Genotypes of 3 significant markers associated with ProtoSol, ADICP_pct and NDFdTrad_48 in top and bottom 20 entries.

## Abbreviations

ADF: acid detergent fiber
aNDF: neutral detergent fiber analyzed with amylase
aNDFom: amylase neutral detergent fiber organic matter
ADICP: acid detergent insoluble crude protein
ADICP_pct: ADICP as a percent of crude protein fraction
CP: crude protein
M6Std_Mperton_48: milk per ton estimated from the “Milk 2006” model using 48-h NDFD (standard method)
M6Trad_Mperton_48: milk per ton estimated from the “Milk 2006” model using 48-h NDFD (traditional method)
NDFdStd_24: NDF digestibility standard in 24 h
NDFdStd_30: NDF digestibility standard in 30 h
NDFdTrad_30: NDF digestibility traditional in 30 h
NDFdTrad_48: NDF digestibility traditional in 48 h
NDFdTrad_120: NDF digestibility traditional in 120 h
NDFdTrad_240: NDF digestibility traditional in 240 h
NDFind_DKC: indigestible NDF (David K. Combs’ protocol)
NDFkd_DKC: NDF digestion rate (David K. Combs’ protocol)
NDFStd_48: NDF digestibility standard in 48 h
NDICP: neutral detergent insoluble crude protein
NFC: non-fibrous carbohydrates
RFQ: relative forage quality index
RFV: relative feed value index
NRC01_Lignin_NEL: Dairy National Research Council 2001 equation for net energy for lactation
ProtoSol: soluble protein
Sugar_ESC: sugar of ethanol soluble carbohydrates
Sugar_WSC: sugar of water soluble carbohydrates
TTNDFD: total tract NDF digestibility
TTNDFD_DKC: total tract NDF digestibility (David K. Combs’ protocol)
uNDF30: 30-h undigested neutral detergent fiber
uNDF120: 120-h undigested neutral detergent fiber
uNDF240: 240-h undigested neutral detergent fiber
