# Supplementary material for: Genome-Wide Association Studies Identifying Multiple Loci Associated With Alfalfa Forage Quality
Source: Front Plant Sci. 2021 Jun 18;12:648192. doi: 10.3389/fpls.2021.648192 (PMC8253570; doi:10.3389/fpls.2021.648192)
Supplement: Supplementary Figure 1 — Population structure with principal component analysis. The top 2 components were used to represent the structure. Clusters 1, 2, and 3 were displayed in green, red and blue respectively. [file Data_Sheet_1.PDF]

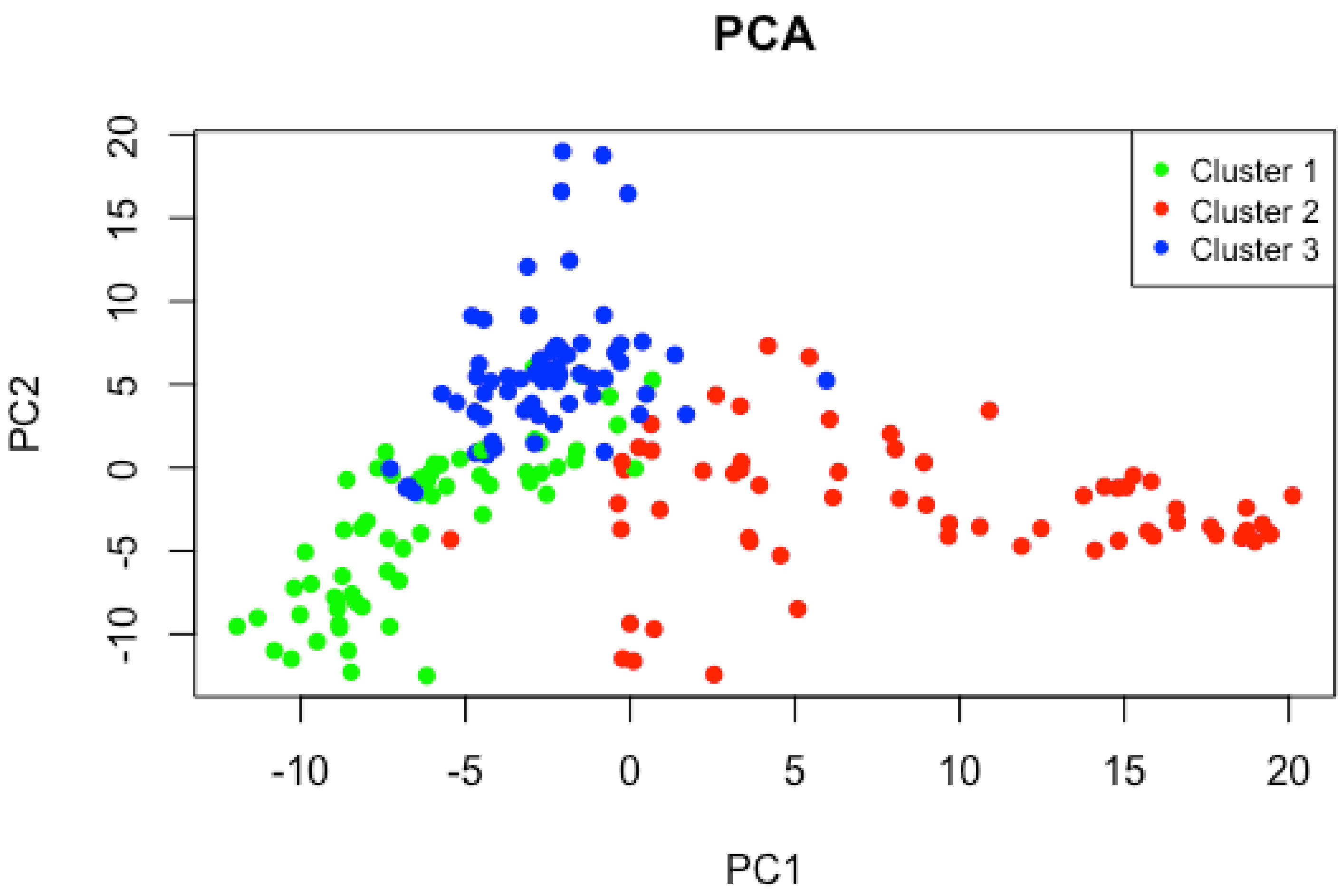

Figure S1 Population structure with principal component analysis. The top 2 components were used to represent the structure. Cluster 1, 2 and 3 were displayed in green, red and blue respectively.

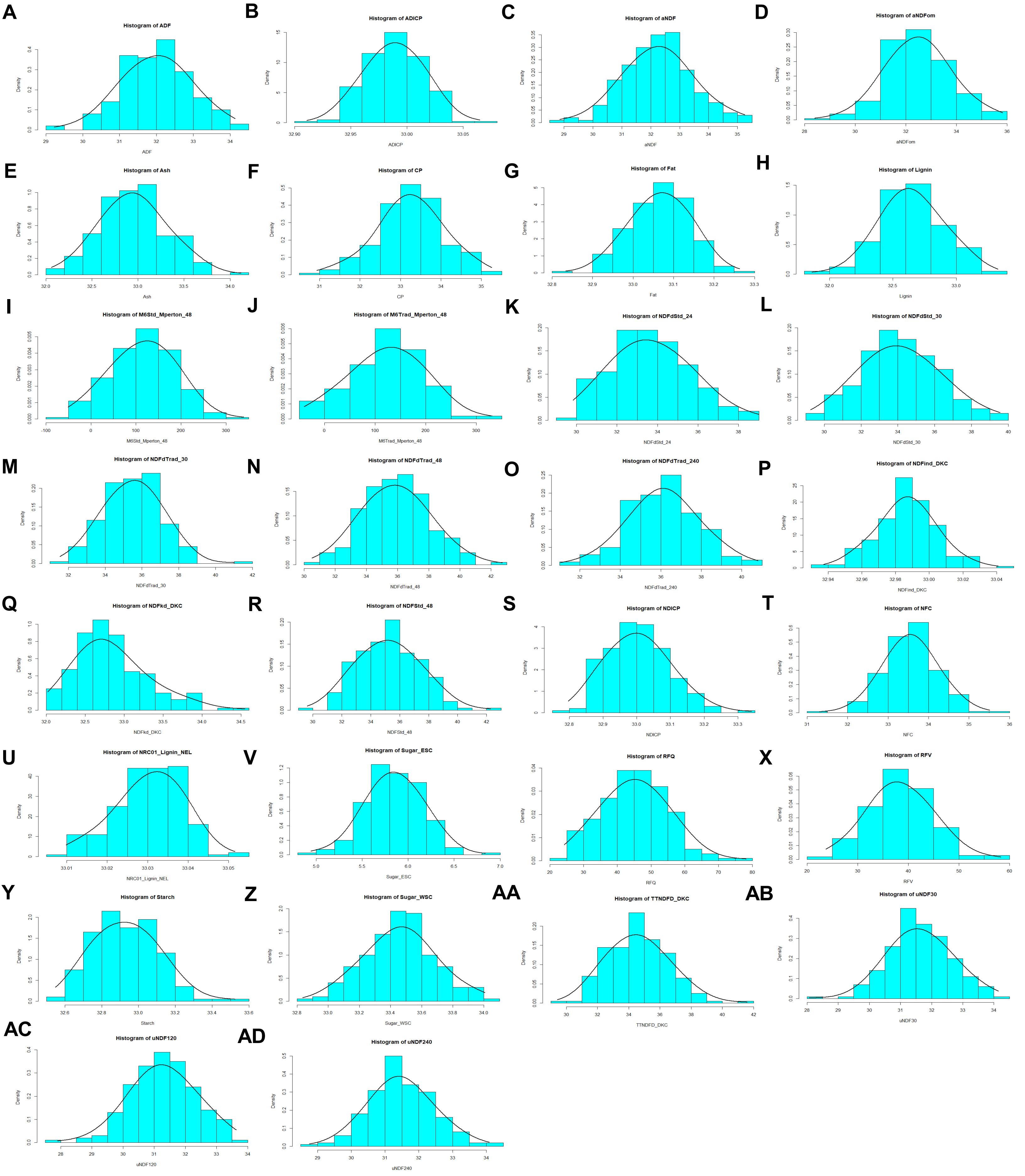

Figure S2 Frequency distribution of different quality traits in alfalfa. (A) ADF, (B) ADICP, (C) aNDF, (D) aNDFom, (E) Ash, (F) CP, (G) Fat, (H) Lignin, (I) M6Std_Mperton_48, (J) M6Trad_Mperton_48, (K) NDFdStd_24, (L) NDFdStd_30, (M) NDFdTrad_30, (N) NDFdTrad_48, (O) NDFdTrad_240, (P) NDFind_DKC, (Q) NDFkd_DKC, (R) NDFStd_48, (S) NDICP, (T) NFC, (U) NRC01_Lignin_NEL, (V) Sugar_ESC, (W) RFQ, (X) RFV, (Y) Starch, (Z) Sugar_WSC, (AA) TTNDFD_DKC, (AB) uNDF30, (AC) uNDF120, (AD) uNDF240.

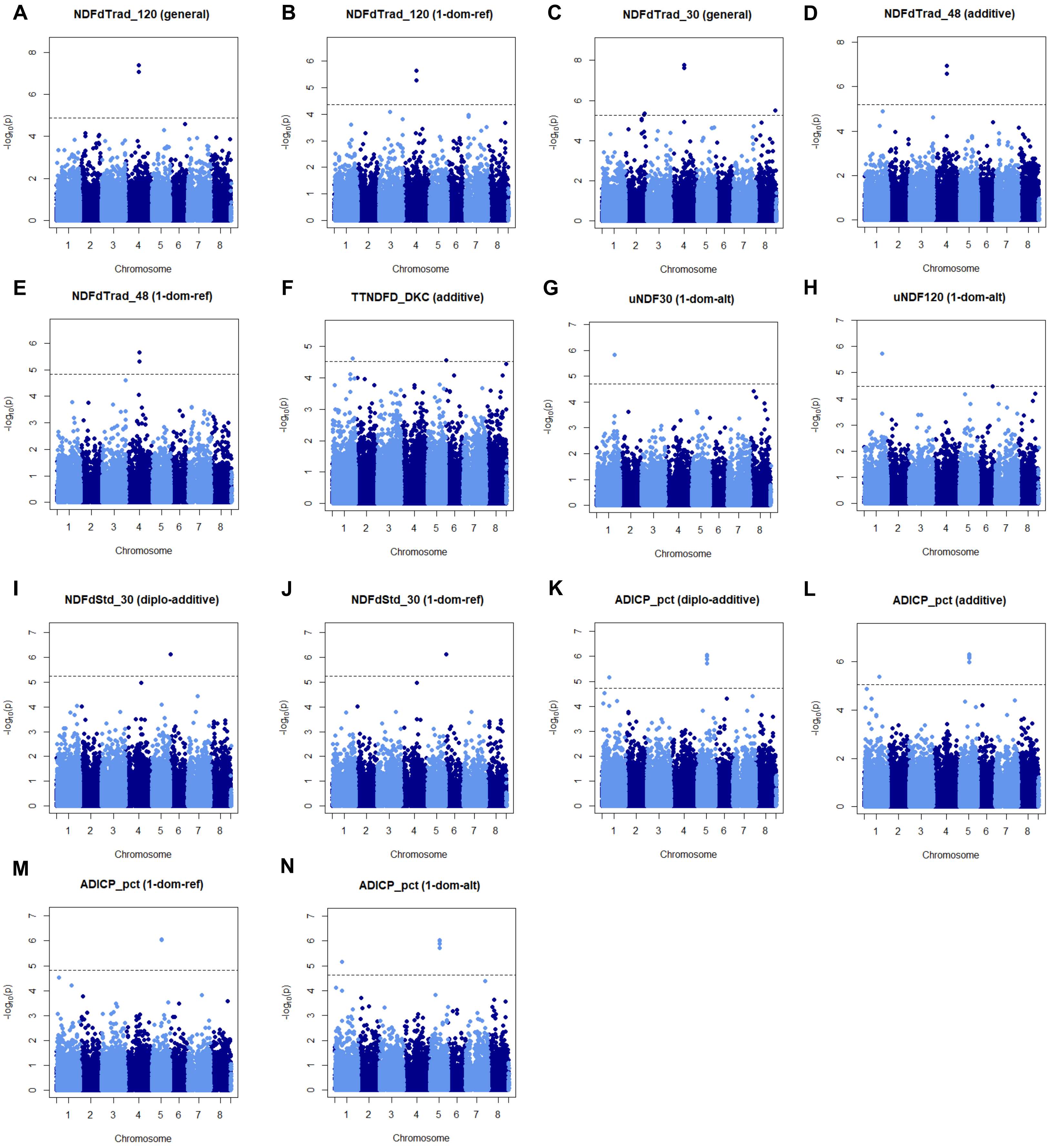

Figure S3 Manhattan plots of significant markers associated with alfalfa quality traits by additional models.

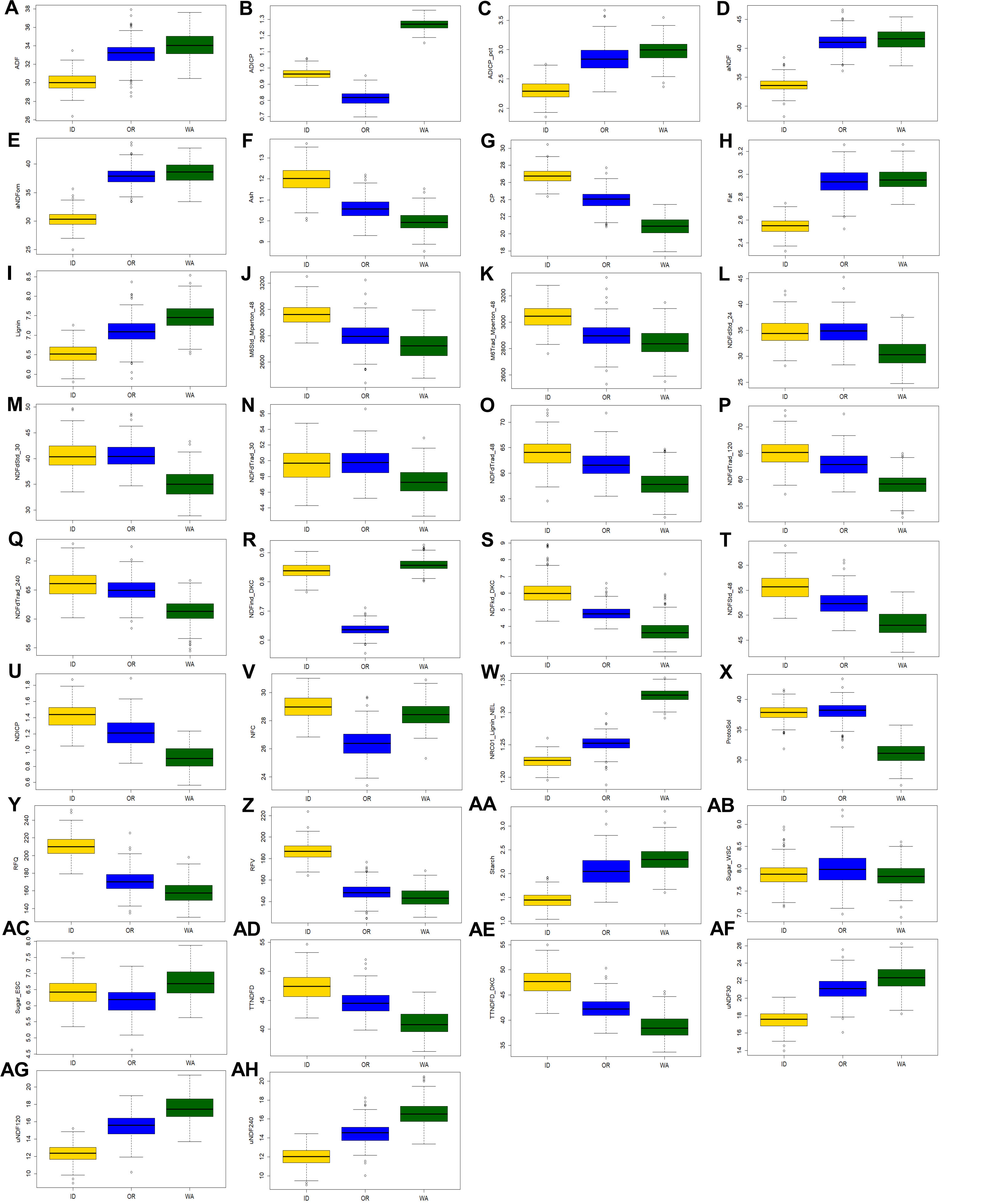

Figure S4 Phenotype distribution of 34 traits in 3 locations. (A) ADF, (B) ADICP, (C)ADICP_pct, (D) aNDF, (E) aNDFom, (F) Ash, (G) CP, (H) Fat, (I) Lignin, (J) M6Std_Mperton_48, (K) M6Trad_Mperton_48, (L) NDFdStd_24, (M) NDFdStd_30, (N) NDFdTrad_30, (O) NDFdTrad_48, (P) NDFdTrad_120 (Q) NDFdTrad_240, (R) NDFind_DKC, (S) NDFkd_DKC, (T) NDFStd_48, (U) NDICP, (V) NFC, (W) NRC01_Lignin_NEL, (X) ProtoSol, (Y) RFQ, (Z) RFV, (AA) Starch, (AB) Sugar_WSC, (AC) Sugar_ESC, (AD) TTNDFD, (AE) TTNDFD_DKC, (AF) uNDF30, (AG) uNDF120, (AH) uNDF240.
